# Muskuloskelettale Pathologien bei Kindern mit infantiler Zerebralparese: ein neues Klassifikationssystem

**DOI:** 10.1007/s00132-026-04776-2

**Published:** 2026-02-11

**Authors:** L. M. Kessling, R. A. van Stralen, J. J. Tolk, H. K. Graham, E. Rutz

**Affiliations:** 1 Abteilung für Orthopädie und Sportmedizin, Erasmus MC – Sophia Kinderkrankenhaus, Erasmus Medical Center, 3015 GD Rotterdam, Niederlande; 2 Murdoch Children’s Research Institute (MCRI), Victoria 3052, Parkville, Australien; 3 Abteilung für Kinderorthopädie, The Royal Children’s Hospital, Victoria 3052, Parkville, Australien; 4 Department of Paediatrics, Bob Dickens Chair, Paediatric Orthopaedic Surgery, The University of Melbourne, Victoria 3010, Parkville, Australien; 5 Medizinische Fakultät, Universität Basel, 4001 Basel, Schweiz; 6 School of Health and Biomedical Sciences, Royal Melbourne Institute of Technology (RMIT University), Melbourne 3000, Melbourne, Australien

**Keywords:** Kind, Kontrakturen, Untere Extremität, Muskuloskelettale Fehlbildungen, Behandlungsergebnis, Child, Contracture, Lower extremity, Musculoskeletal abnormalities, Treatment outcome

## Abstract

**Hintergrund:**

Die infantile Zerebralparese (ICP) ist eine der häufigsten Ursachen für körperliche Beeinträchtigungen im Kindesalter. Während das Gross Motor Function Classification System (GMFCS) die motorischen Fähigkeiten beschreibt, fehlte bisher eine einheitliche Klassifikation für muskuloskelettale Pathologien. Das vorgestellte 4‑stufige System – entwickelt auf Basis des Mercer-Rang-Modells – dient der Beschreibung der Krankheitsprogression der unteren Extremitäten und unterstützt Diagnose, Therapieplanung und Forschung.

**Stadium 1:**

Muskuläre Hypertonie: Von der Geburt an bis zu einem Alter von 6 Jahren dominieren Spastizität und verzögerte Motorikentwicklung; Kontrakturen sind selten. Frühintervention und Spastiktherapie (z. B. Botulinumtoxin) stehen im Vordergrund.

**Stadium 2:**

Kontrakturen: Zwischen 4 und 12 Jahren führen Längenunterschiede zwischen Muskeln und Knochen zu Bewegungseinschränkungen. Operative Muskel- oder Sehnenverlängerungen können notwendig werden.

**Stadium 3:**

Knöcherne Deformitäten: Gleichzeitig entstehen knöcherne Fehlstellungen wie femorale Antetorsion oder Pes valgus mit Kontrakturen, kombinierte Multilevel-Eingriffe (SEMLS) sind oft indiziert.

**Stadium 4:**

Dekompensierte Pathologien: Nach der Pubertät treten irreversible Fehlstellungen und Gelenkdegenerationen auf. Eingriffe dienen meist der Schmerzreduktion oder Stabilisierung (z. B. Arthrodese).

**Schlussfolgerung:**

Das System fördert das Bewusstsein für die Krankheitsprogression, erleichtert die Wahl stadiengerechter Therapien und kann Über- oder Unterbehandlungen vermeiden. Frühzeitige Interventionen sind entscheidend, um Dekompensationen vorzubeugen und die funktionellen Ergebnisse zu verbessern.

**Graphic abstract:**

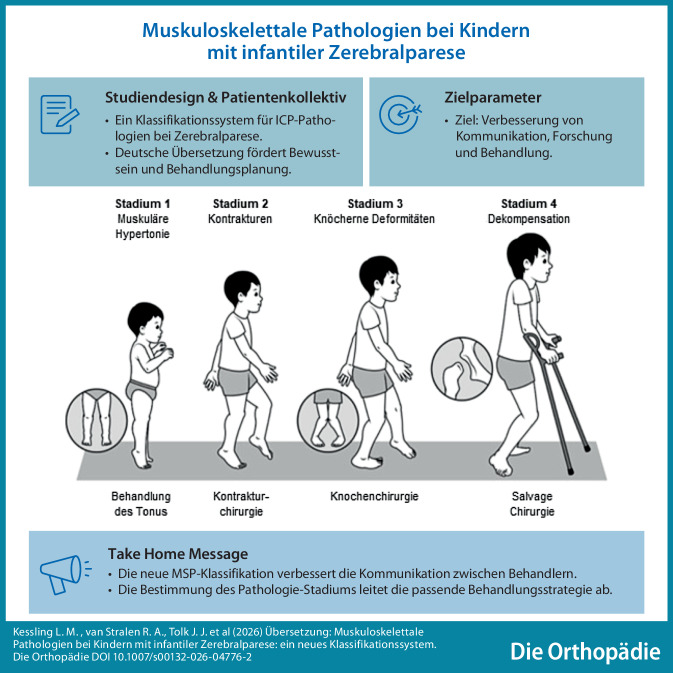

## Abstract (en)

**Background:**

Cerebral palsy (CP) is one of the most common causes of physical disability in childhood. While the Gross Motor Function Classification System (GMFCS) describes motor abilities, a unified classification for musculoskeletal pathologies was lacking. The newly proposed four-stage system—based on the Mercer Rang model—describes the progression of lower limb pathology and supports diagnosis, treatment planning, and research.

**Stage 1:**

Hypertonia: From birth to about 6 years, spasticity and delayed motor development predominate; contractures are rare. Early intervention and spasticity management (e.g., botulinum toxin) are the focus.

**Stage 2:**

Contractures: Between the ages of 4 and 12 years, discrepancies between muscle-tendon length and bone growth cause a reduced range of motion. Surgical muscle or tendon lengthening may be indicated.

**Stage 3:**

Bony deformities: Bony deformities such as increased femoral anteversion or pes valgus occur simultaneously with soft tissue contractures; rotational osteotomies and combined multilevel surgeries (SEMLS) are often required.

**Stage 4:**

Decompensated pathology: After puberty, irreversible deformities and joint degeneration develop. Surgery usually aims at pain reduction or stabilization (e.g., arthrodesis).

**Conclusion:**

This classification raises awareness of disease progression, helps select stage-appropriate treatments, and may prevent over- or undertreatment. Early recognition and intervention are crucial to avoid decompensation and improve long-term musculoskeletal and functional outcomes.

## Einleitung

Die Infantile Zerebralparese (ICP) ist eine der häufigsten Ursachen für körperliche Beeinträchtigungen im Kindesalter und betrifft schätzungsweise 2–4 von 1000 Lebendgeborenen [5]. Zur Einteilung der motorischen Fähigkeiten von Kindern mit ICP wird weltweit das Gross Motor Function Classification System (GMFCS) verwendet [9]. Dieses Klassifikationssystem liefert wertvolle Informationen über die funktionellen Fähigkeiten des Kindes, unterstützt die Kommunikation zwischen Behandlern und Eltern und hilft, realistische Erwartungen im Hinblick auf Verlauf und Therapieplanung zu formulieren.

**Abb. 1:**
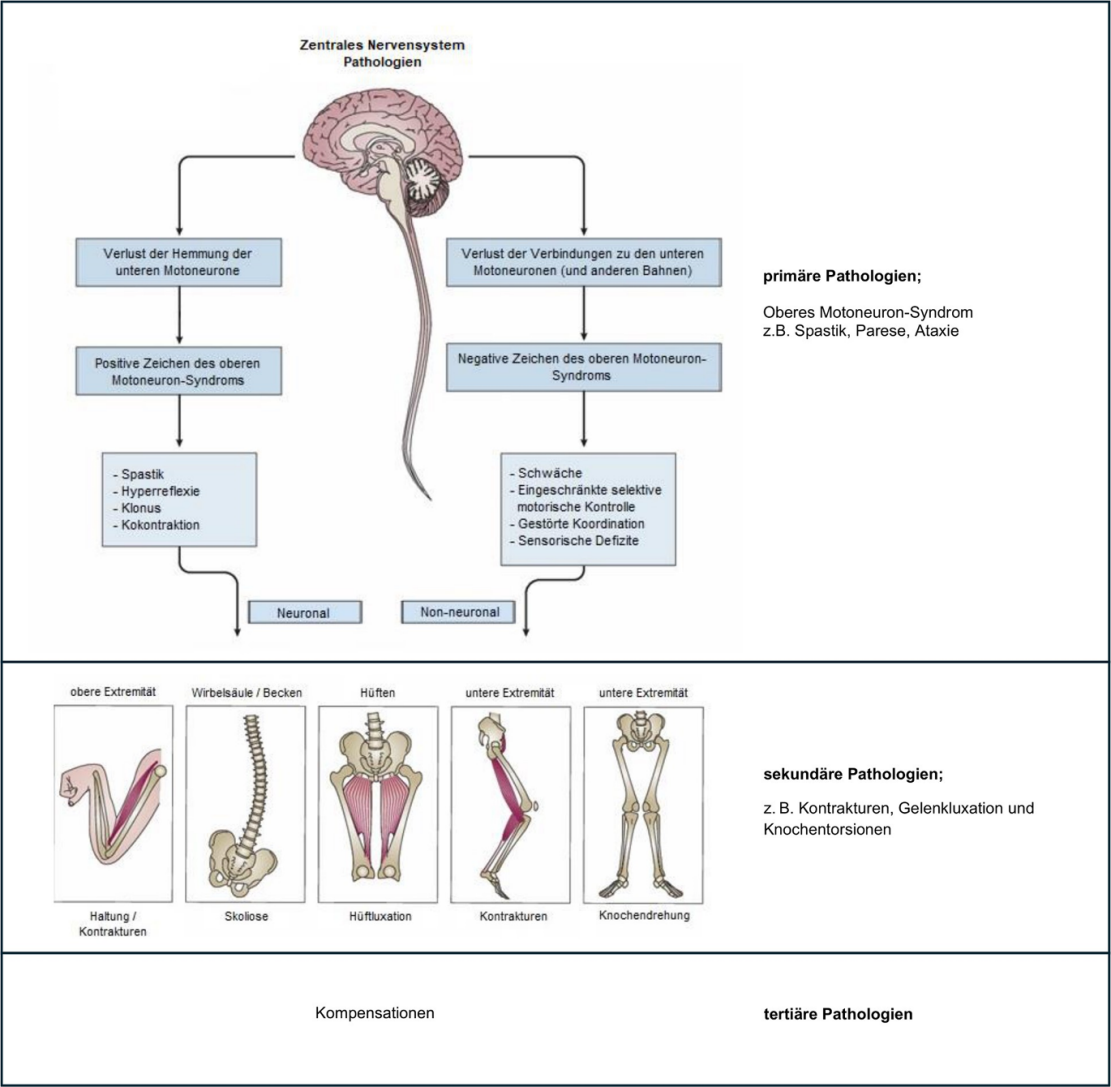
Darstellung des klassischen Modells der 3 Pathologiestufen (primär, sekundär, tertiär) bei Kindern mit Zerebralparese

Traditionell wird die Entwicklung muskuloskelettaler Pathologien bei Kindern mit Zerebralparese in 3 Stufen unterteilt. Primäre Pathologien resultieren direkt aus der nichtprogredienten Schädigung des sich entwickelnden zentralen Nervensystems (ZNS) und sind von Beginn an vorhanden. Sie sind neurologischer Natur und umfassen Spastik (erhöhter Muskeltonus), Muskelschwäche (Paresen), eingeschränkte selektive motorische Kontrolle, Ataxie (Koordinationsstörungen) sowie Gleichgewichtsstörungen. (Abb. 1; [6]) Diese Manifestationen spiegeln die Unfähigkeit des Gehirns wider, motorische Signale korrekt zu senden oder zu verarbeiten. Sekundäre Pathologien entwickeln sich im Verlauf des Wachstums als indirekte Folge der primären neurologischen Defizite. Sie sind oft progressiv und entstehen durch abnorme Belastungen oder Bewegungsmangel. Typische Beispiele sind Muskel-Sehnen-Kontrakturen, Subluxationen oder Luxationen der Hüfte sowie torsionale Knochenfehlstellungen, wie z. B. Femuranteversion. Diese Veränderungen in Muskeln und Knochen sind nicht angeboren, sondern entstehen als Reaktion auf Spastik oder Muskelschwäche. Tertiäre Pathologien bezeichnen schließlich kompensatorische Mechanismen oder „Coping-Strategien“, die die Betroffenen entwickeln, um primäre und sekundäre Einschränkungen auszugleichen, häufig im Rahmen des Gehens. Beispiele sind übermäßige Hüftumkreisungen, Vaulting (Aufzehenstellung des gegenüberliegenden Fußes) oder Rumpfneigung zur Aufrechterhaltung des Gleichgewichts. Alternativ wird tertiäre Pathologie in einigen Studien auch als persistierende aktive biologische Mechanismen wie Entzündungen oder oxidativer Stress definiert, die die Regeneration des Gehirns über Monate oder Jahre nach der initialen Verletzung verhindern. Dieses klassische Modell wird insbesondere in der US-amerikanischen Neurologie oft in den 3 Kategorien neurologische Defizite, muskuloskelettale Pathologie und kompensatorische Strategien dargestellt (Abb. 1; [6]).

Trotz der weit verbreiteten Darstellung muskuloskelettaler Pathologien bei Kindern mit Zerebralparese in den 3 klassischen Stufen gibt es bislang keine allgemein akzeptierte Klassifikation dieser Pathologien. Abgesehen von der schematischen Gruppierung durch Dr. Mercer Rang existiert kein etabliertes System, das Kinder zur Unterstützung von Behandlungsentscheidungen oder als Grundlage für klinische Forschung einordnet [10].

Im Jahr 2021 entwickelte ein australisches Forschungsteam auf Basis des Modells von Mercer Rang ein 4‑stufiges Klassifikationssystem zur Beschreibung der muskuloskelettalen Pathologien der unteren Extremitäten bei Patienten mit ICP, das vom Kleinkindalter bis ins Erwachsenenalter anwendbar ist [3, 7].

Ziel dieses Systems ist es, Kommunikation, Ausbildung und wissenschaftliche Forschung zu fördern, die Indikationsstellung geeigneter Interventionen zu erleichtern, iatrogene Schäden zu reduzieren und vor allem die langfristige muskuloskelettale Gesundheit sowie das funktionelle Ergebnis von Jugendlichen und jungen Erwachsenen mit ICP zu optimieren.

Eine deutsche Übersetzung dieser Klassifikation, die von allen beteiligten Behandelenden verwendet werden kann, kann das Bewusstsein für die Entstehung und Entwicklung muskuloskelettaler Pathologien bei Kindern mit ICP erhöhen und zur Planung geeigneter Behandlungsstrategien beitragen.

## Klassifikation

**Abb. 2:**
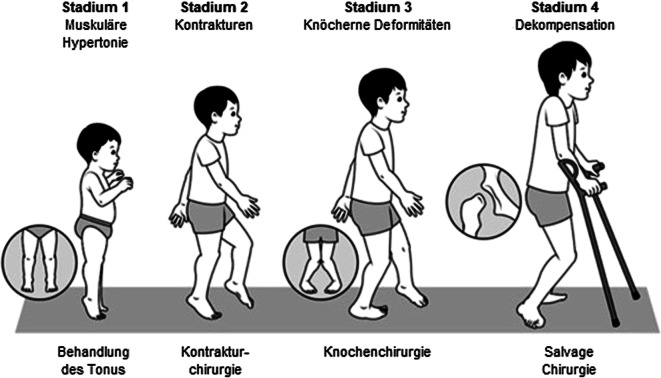
Die verschiedenen Stadien der muskuloskelettalen Pathologien (Gross) bei Kindern mit infantiler Zerebralparese im Verlauf der Entwicklung. Es besteht häufig eine Überlappung zwischen Stadium 2 und 3

Eine Übersicht des Verlaufs muskuloskelettaler Pathologien ist in Abb. 2 dargestellt. Die übersetzte Klassifikation ist in Tab. 1 wiedergegeben. Es ist ausdrücklich zu betonen, dass die bei den einzelnen Stadien genannten Altersangaben lediglich orientierenden Charakter haben und weder feste Altersgrenzen noch eine eindeutige Zuordnung der Stadien zu bestimmten Alterskategorien implizieren, da grundsätzlich jedes Stadium in allen Altersbereichen auftreten kann.

**Tab. 1:** Muskuloskelettale Pathologie bei Kindern mit spastischer ICP, differenziert nach anatomischem Niveau.

Niveau	Stadium 1 Muskuläre Hypertonie Ab Geburt bis zum Alter von 4–6 Jahren*	Stadium 2 Kontrakturen Alter von 4–12 Jahren	Stadium 3 Knöcherne Deformitäten Alter von 4–12 Jahren	Stadium 4 Dekompensation Von 10 Jahren bis Erwachsenenalter
Hüfte	Haltung in Flexion/Adduktion Klinisch: Scherengang	Flexions‑/Adduktionskontrakturen	Zugenommener FNA ( 25°, Hüfte IR 2 Standardabweichung Innenrotation auf 3DGA) Zugenommener MI Azetabulumdysplasie	Deformität des Femurkopfes Azetabuläre Deformität Verlust von Gelenkknorpel Arthrose
Knie	Spastische Knieflexion Hamstring-Spastizität Vollständige Knieextension und teilweise Hyperextension	Hamstring-Kontraktur Zugenommener Poplitealwinkel Vollständige Knieextension oder Flexionskontraktur des Knies 10°	Arthrogene Bewegungseinschränkung, Flexionskontraktur des Knies 20° Fehlstellung: femorale Anteversion und externe tibiale Torsion Genu valgum/Genu varum	Patella alta Flexionskontraktur des Knies 20° Patellafraktur/Avulsion Arthrose
Sprunggelenk	Dynamischer Spitzfuß Sprunggelenk korrigiert bis 0° mit Knie in Extension	Spitzfuß nicht zu korrigieren Sprunggelenk DF 0° mit Knie in Extension Untersuchung in Narkose kann für eine bessere Beurteilung hilfreich sein	*Tibiale Torsion:* – externe tibiale Torsion (ETT) 20° – interne tibiale Torsion (ITT) 10°	Hackenfuß, zu lange Achillessehne Deformität des Talus Arthrose Beinlängenunterschied 2,0 cm am Ende des Wachstums
Fuß	Flexible Varus- oder Valgus-Fußposition	Teilweise flexibler/rigider Varus mit muskulärer Dysbalance und/oder Kontrakturen	Rigider Equinovarus, Equinocavovarus Pes valgus mit LAD Bestätigt mit Röntgenbildern und Pedobarographie	Schwielen und Dekubiti Stressfrakturen der Metatarsalia Deformierte Tarsalia Arthrose
Therapie	*Behandlung des Tonus:* – orale Tonusmedikation – Botulinum Toxin A (BoNT-A) – selektive dorsale Rhizotomie – intrathekal Baclofen – AFO und Physiotherapie	*Kontrakturchirurgie:* – Weichteilchirurgie – Sehnenverlängerung – Sehnentransfer – AFO und Physiotherapie	*Knochenchirurgie:* – Osteotomien und gelenksstabilisierende Eingriffe – oft inklusive Weichteileingriffen; – SEMLS/MLS – Wachstumssteuerung bei Flexionskontraktur und Beinlängenunterschied – AFO und Physiotherapie	*Salvage-Therapie:* – komplexe Rekonstruktionen, wie extendierende Femurosteotomien, Kürzung der Patellasehne, PAO – Arthrodesen und Prothetik – Hilfsmittel und Rollstuhlapparaturen – Anpassen der Umgebung – Physiotherapie und Ergotherapie

*AOT* femorale Anteversion (FNA), *MLS* „multi level surgery“, *MI* Migrationsindex, *PAO* periazetabuläre Osteotomie, *IR* Innenrotation, *DF* Dorsalflexion, *LAD* „lever arm deformity“, *AFO* „ankle-foot orthoses“, *SEMLS* „single-event multi level surgery“

*Die in dieser Tabelle genannten Altersangaben dienen als Richtwerte für den zeitlichen Rahmen und stellen keine strikten Grenzwerte dar

### Stadium 1: Muskuläre Hypertonie – von der Geburt bis etwa 4–6 Jahre

Stadium 1 umfasst Kinder mit Zerebralparese von der Diagnosestellung bis etwa zum 6. Lebensjahr und entspricht der primären Pathologie nach der traditionellen Auffassung [4, 6]. Die Hauptprobleme sind muskuläre Hypertonie (Spastizität, Dystonie und gemischte Bewegungsstörungen) sowie eine verzögerte Entwicklung der grobmotorischen Fähigkeiten. In dieser Phase bestehen nur selten Kontrakturen und orthopädische Operationen sind in der Regel nicht erforderlich [6].

Die Behandlung konzentriert sich auf Frühintervention zur Förderung der grobmotorischen Entwicklung, typischerweise durch Physiotherapie, Behandlung der Spastiken und den Einsatz von Fußorthesen und Hilfsmitteln. Fokale Spastizität, beispielsweise bei Spitzfuß, kann mit Botulinum-Neurotoxin Typ A (BoNT-A) behandelt werden. Typische Veränderungen auf den verschiedenen anatomischen Niveaus sind in Tab. 1 dargestellt. Nach etwa 5 Jahren nimmt die Spastizität ab, während Kontrakturen zunehmen [4].

### Stadium 2: Kontrakturen – etwa von 4–12 Jahre

Bei Kindern mit ICP nimmt der Bewegungsumfang der Gelenke aufgrund einer zunehmenden Diskrepanz zwischen der Länge der Muskel-Sehnen-Einheiten und dem Wachstum der angrenzenden Knochen ab. In dieser Phase werden häufig Muskelkontrakturen festgestellt, die das Gehen und die Funktion beeinträchtigen können. Dies ist das erste Anzeichen für die Entwicklung der sekundären Pathologie in der ursprünglichen Klassifikation [6].

Die Behandlung in Stadium 2 zielt auf die Verlängerung von Muskeln und Sehnen ab und kann operative Maßnahmen beinhalten, z. B. Muskelverlängerungen oder Sehnentranspositionen.

Konkrete Grenzwerte für Kontrakturen existieren nicht, doch gilt eine Dorsalflexion des Sprunggelenks unter Neutralstellung (bei gestrecktem Knie) sowie jede Form fester Flexionskontraktur von Knie oder Hüfte als klinisch relevant.

**Abb. 3:**
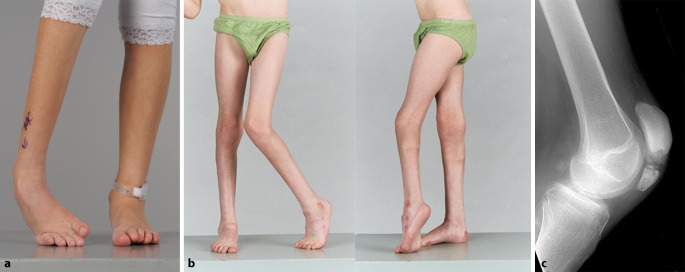
Beispiele für Pathologien der Stadien 2, 3 und 4. Aus [3], mit freundl. Genehmigung, **a** Stadium 2: Pes equinovarus bei einem 7‑jährigen Mädchen mit infantiler Zerebralparese (ICP; Gross Motor Function Classification System [GMFCS] II). Fixierte Kontraktur des M. gastrocnemius, M. soleus und M. tibialis posterior. **b** Stadium 3: 10-jähriger Junge mit ICP (GMFCS II), asymmetrische Diplegie. Vorhandene Spitzfußdeformität und femorale Torsion, *links*: Vorderansicht, *rechts*: Seitenansicht. **c** Stadium 4: Laterale Röntgenaufnahme des Knies eines 15-jährigen Jungen mit ausgeprägtem Crouch-Gait. Stressfraktur des Patellaunterpools infolge chronischer Überlastung des Streckapparats

Muskel-Sehnen-Verlängerungen auf einer oder mehreren Ebenen können in diesem Stadium indiziert sein; dabei müssen Spastizität, Muskelschwäche und Wachstumseffekte berücksichtigt werden ([2, 12]; Abb. 3a; Tab. 1).

### Stadium 3: Knochendeformitäten – etwa von 4–12 Jahren

Die meisten Kinder mit ICP, die Kontrakturen entwickeln, zeigen gleichzeitig knöcherne Deformitäten. Eine erhöhte femorale Antetorsion (FNA) ist bei den meisten Kindern mit ICP bereits bei Geburt vorhanden und scheint nicht primär durch Spastizität bedingt zu sein. Eine FNA von mehr als 25° gilt als pathologisch und dient als Schwellenwert für die Operationsindikation [8].

Externe Tibiatorsion (ETT) entwickelt sich graduell sowohl bei Kindern mit ICP als auch bei gesund entwickelten Kindern. Gelenkinstabilitäten wie Hüftdysplasie treten bei gehfähigen Kindern seltener und milder auf. Mittelfußinstabilität und Pes valgus sind häufig mit einer Spitzfußkontraktur kombiniert [6] (Abb. 3b; Tab. 1).

In Stadium 3 können Rotationsosteotomien und Eingriffe zur Gelenkstabilisierung indiziert sein, meist im Rahmen einer kombinierten „single-event multilevel surgery“ (SEMLS) [2, 12].

Die in den Stadien 2 und 3 beschriebenen Pathologien fallen unter die sekundäre Pathologiegruppe der traditionellen Klassifikation.

### Stadium 4: Dekompensierte Pathologien – ab etwa 10 Jahren bis ins Erwachsenenalter

Dekompensation bedeutet, dass die muskuloskelettalen Pathologien soweit fortgeschritten sind, dass eine Wiederherstellung der optimalen Gelenk- und Muskelfunktion nicht mehr möglich ist. Dies tritt meist nach dem pubertären Wachstumsschub auf, kann aber auch früher vorkommen [6].

Kennzeichnend sind schwere Gelenkkontrakturen und Knochenverformungen, kombiniert mit Muskel-Sehnen-Kontrakturen, Muskelschwäche und Hypertonie.

Chirurgische Maßnahmen in diesem Stadium dienen meist der Salvage-Chirurgie und sind nicht primär rekonstruktiv. Dazu zählen Gelenkversteifungen (Arthrodesen) oder -ersatz infolge von Arthrose. Bei jüngeren Kindern mit noch flexiblen Fehlstellungen wie Equinovarus oder Pes valgus sind gelenkerhaltende Eingriffe oft erfolgreich; bleiben diese jedoch unbehandelt, entstehen rigide Deformitäten der Fußwurzelknochen, die Arthrodesen erforderlich machen ([1, 11]; Tab. 1; Abb. 3c).

Die in Stadium 4 beschriebenen Pathologien fallen unter die klassische tertiäre Pathologie.

## Diskussion

Diese neue Klassifikation bietet ein wertvolles Instrument, um die jeweils geeignete Behandlungsstrategie in Abhängigkeit vom Stadium der muskuloskelettalen Pathologien zu bestimmen.

Es ist zu beachten, dass Überschneidungen zwischen den Stadien – insbesondere zwischen Stadium 2 und 3 – häufig vorkommen. Die meisten Kinder mit ICP zeigen eine Kombination aus Weichteilkontrakturen, knöchernen Torsionsproblemen, Restspastizität und Muskelschwäche (Abb. 3a und b).

Die Klassifikation erhöht zudem das Bewusstsein für das aktuelle Erkrankungsstadium und kann so Über- oder Unterbehandlungen verhindern, etwa durch sogenannte „category errors“, d. h. die Behandlung einer Pathologie eines bestimmten Stadiums mit einer für ein anderes Stadium vorgesehenen Strategie [3].

Ein Beispiel wäre eine isolierte Hamstring-Verlängerung bei rigider Knieflexionsdeformität infolge degenerativer Gelenkveränderungen, hier wäre eine solche Weichteilmaßnahme unzureichend.

Frühzeitige, minimalinvasive Interventionen, etwa zur Prävention der Hüftmigration, unterstreichen die Bedeutung eines rechtzeitigen Handelns. Durch frühe Erkennung und Intervention kann das Behandlungsteam eine Dekompensation vermeiden und weniger belastende Verfahren anwenden. Entscheidend ist die frühzeitige Identifikation der Progression zu Stadium 2 oder 3.

Diese Klassifikation bietet somit ein praxisnahes Instrument, um die Entwicklung muskuloskelettaler Veränderungen bei Kindern mit ICP systematisch zu beschreiben, wenngleich sie eine vereinfachte Darstellung einer oft komplexen klinischen Realität bleibt [3].

## Fazit für die Praxis

- Die Einteilung nach der neuen „Musculoskeletal Pathology“(MSP)-Klassifikation kann die Kommunikation zwischen Behandlern verbessern.
- Sie hilft, das Stadium der Pathologieentwicklung zu bestimmen und daraus eine geeignete Behandlungsstrategie abzuleiten.

## Data Availability

Die Weitergabe von Daten ist für diesen Artikel nicht relevant, da in dieser Studie keine neuen Daten erstellt oder analysiert wurden.

## Abkürzungsverzeichnis

AFO: Ankle-foot orthoses
BoNT-A: Botulinum-Neurotoxin Typ A
DF: Dorsalflexion
ETT: Externe tibiale Torsion
FNA: Femorale Anteversion
GMFCS: Gross Motor Function Classification System
ICP: Infantile Zerebralparese
IR: Innenrotation
ITT: Interne tibiale Torsion
MI: Migrationsindex
MLS: Multi level surgery
MSP: Musculoskeletal Pathology
LAD: Lever arm deformity
PAO: Periazetabuläre Osteotomie
SEMLS: Single-event multi level surgery
ZNS: Zentralen Nervensystems
3dGA: 3D Ganganalyse
